# Child-oriented word associations improve models of early word learning

**DOI:** 10.3758/s13428-022-01790-y

**Published:** 2022-03-07

**Authors:** Christopher R. Cox, Eileen Haebig

**Affiliations:** 1 Department of Psychology, Louisiana State University, Baton Rouge, LA USA; 2 Department of Communication Sciences and Disorders, Louisiana State University, Baton Rouge, LA USA

**Keywords:** Language, Learning, Semantics, Word associations

## Abstract

How words are associated within the linguistic environment conveys semantic content; however, different contexts induce different linguistic patterns. For instance, it is well known that adults speak differently to children than to other adults. We present results from a new word association study in which adult participants were instructed to produce either unconstrained or child-oriented responses to each cue, where cues included 672 nouns, verbs, adjectives, and other word forms from the McArthur–Bates Communicative Development Inventory (CDI; Fenson et al., 2006). Child-oriented responses consisted of higher frequency words with fewer letters, earlier ages of acquisition, and higher contextual diversity. Furthermore, the correlations among the responses generated for each pair of cues differed between unconstrained (adult-oriented) and child-oriented responses, suggesting that child-oriented associations imply different semantic structure. A comparison of growth models guided by a semantic network structure revealed that child-oriented associations are more predictive of early lexical growth. Additionally, relative to a growth model based on a corpus of naturalistic child-directed speech, the child-oriented associations explain added unique variance to lexical growth. Thus, these new child-oriented word association norms provide novel insight into the semantic context of young children and early lexical development.

Early language acquisition involves a dynamic interplay between children and their environment that changes as they develop (McClelland et al., 2010; Smith et al., 2018; Smith & Thelen, 2003; Thelen & Smith, 1996). Previous studies have demonstrated that the number and diversity of words in a child’s environment predicts language outcomes (Hart & Risley, 1995; Hoff, 2003; Huttenlocher et al., 2010). Furthermore, how words relate within the environment—their associative and semantic structure—influences early language acquisition. Words that are central in the environment or are related to many words that are currently known are learned earlier (Dubossarsky et al., 2017; Hills, 2013; Hills et al., 2009). Thus, investigations of early word learning rely on accurate descriptions of both the composition and semantic content of the child linguistic environment. The current study evaluates a method of measuring child-oriented language environments based on adult-generated word associations and tests whether these data strengthen computational models of early word learning.

## Child-directed speech differs from adult-directed speech

Child-directed speech is grammatically and phonologically simpler than speech directed at other adults. It consists of more nouns than pronouns or verbs (Ferguson, 1964; Hayes & Ahrens, 1988; Soderstrom, 2007) and has more word repetitions (Hills, 2013), all delivered with unique prosody. Child-directed speech also has distinctive distributional qualities: child-directed speech is more likely to present highly associated words in close proximity and favor words that appear in many contexts (i.e., words with high contextual diversity; Hills, 2013). Relatedly, how words co-occur helps shape their meaning and influences word processing in young children (e.g., Willits et al., 2013). Such statistical linguistic properties motivate natural language processing (NLP) techniques that estimate semantic structure from literary and linguistic corpora. While child-directed speech has been a focus of research for decades, our understanding of the semantic content of child-directed speech is limited. An emerging body of research is beginning to examine the unique features of the visual world in which young children interact and learn. For instance, young children experience statistical regularities in their visual fields that have meaningful implications for early lexical development (e.g., Clerkin et al., 2017). Additionally, like child-directed speech, adults also influence young children’s visual learning environments to promote visually rich learning experiences (McQuillan et al., 2020). The semantic environment is multimodal and consists of associations of all kinds, not just the structure of spoken language.

Due to limitations on the kinds of tasks young children can engage with, their small (or nonexistent) productive vocabulary, and the high cost associated with transcribing recordings, existing data that provides insight into the child semantic environment is limited. This has led researchers to substitute estimates of the *adult* semantic environment as a proxy for the child semantic environment, despite the general appreciation that there may be important differences. For example, previous studies have found that the semantic structure conveyed by adult free-association norms are predictive of word learning patterns in children younger than 30 months of age (e.g., Bilson et al., 2015; Hills et al., 2009; Steyvers & Tenenbaum, 2005).

However, it is well established that children preferentially attend to speech that is directed towards them, and early language learning is disproportionately influenced by child-directed speech rather than language that is merely overheard (Shneidman & Goldin-Meadow, 2012). Therefore, unconstrained free-association norms likely yield estimates of semantic structure that differ in important ways from the environment that children develop in and learn from. Those studying child language acquisition appreciate that more age-appropriate semantic norms may be critical to enhancing our understanding of early lexical knowledge (e.g., Dubossarsky et al., 2017). In the current study, we evaluate whether this gap may be reduced by appealing to word association data collected from adults after establishing a child-centered context.

## Estimating semantic structure from observable behavior

Semantic structure cannot be observed directly, so it is inferred from behavior that can be. For instance, word associations generated by participants that are presented with a cue word and asked to report the first related word or a set of words that come to mind reflect multiple kinds of similarity that can be understood as semantic (De Deyne & Storms, 2008; Nelson et al., 1998; Nelson et al., 2000). The University of South Florida (USF) Free Association Norms (Nelson et al., 2004) have been used to estimate the relationships among early-acquired words as an associative network. Prior work indicates that networks constructed from adult-centric word association norms can predict lexical growth better than random growth models (e.g., Hills et al., 2009) and models that are informed by lexical metrics such as word frequency and phonotactic probability (Bilson et al., 2015; Hills et al., 2009; Steyvers & Tenenbaum, 2005). More recently, the Small World of Words project (SWOW) published word associations for over 12,000 English cue words under a three-response protocol (De Deyne et al., 2019). The three-response protocol supports semantic networks that are more predictive of adult judgments of semantic relatedness and lexical access and implies a more densely connected lexical network (De Deyne et al., 2013; De Deyne, Perfors, & Navarro, 2016a).

Another common way of estimating semantic structure within large linguistic environments is by applying natural language processing to large text corpora of published writing or transcriptions from other media. While children produce precious little content of this kind themselves, thousands of transcripts from adult–child interactions have been curated and shared via the Child Language Data Exchange System (CHILDES; MacWhinney, 2000). These interactions were collected during various tasks including toy play, book reading, and unstructured conversations that were recorded in the home or lab environment.

Despite being orders of magnitude smaller than text corpora commonly used to model adult semantic structure, associative structure present in CHILDES (defined by aggregating word co-occurrence statistics) can also be used to define networks that are able to predict child word learning patterns (Hills et al., 2010; Jimenez & Hills, 2017). Basing models of the child semantic environment on transcripts available through CHILDES has the advantage of deriving directly from samples of child language environments (albeit importantly limited ones). Computation models of the North American English language sample in CHILDES are capable of extracting a remarkable amount of thematic and taxonomic semantic structure (Huebner & Willits, 2018).

However, language transcripts have the disadvantage of being a less direct measure of semantic association because co-occurrence statistics are influenced by the syntax of the language (not just its content). Additionally, many words that two- or three-year-old children would be expected to know are spoken relatively rarely in CHILDES—even familiar words with early ages of acquisition (AoA) such as those included on the McArthur–Bates Child Developmental Inventory vocabulary checklists. For instance, within the adult utterances in the CHILDES transcripts, some words were produced frequently but have late AoA (“we” and “think” occur 32,417 and 17,786 times, respectively, in our sample of CHILDES transcripts but are not typically produced until month 30), while others are produced infrequently yet have early AoA (“banana” and “bye” are produced 720 and 1789 times, respectively, in our sample of CHILDES transcripts but are typically produced by month 16). It is possible that some of these surprisingly low frequencies in the CHILDES database are related to the types of tasks that the adults were asked to engage in while the adult–child language samples were recorded. As a result, although valuable, CHILDES likely only offers us a limited picture of the child’s language environment relative to the range of linguistic input that a child experiences across various contexts throughout a typical day (Tamis-LeMonda et al., 2017). Notably, the vast majority of these language samples were collected before tools like LENA enabled child language researchers to collect day-long recordings to estimate a child’s language environment. The Language ENvironment Analysis (LENA) tool is a wearable device that audio-records and automatically analyzes a child’s vocalizations and the language that the child hears. Although highly useful, it is important to note that LENA only quantifies the number of words and conversational turns; it does not transcribe the actual words that are recorded, which would require significant transcription efforts (LENA Research Foundation, 2015).

Lastly, while human development and the full complexity of the environment in which a child acquires language may interact to help compensate for such word frequency effects (Smith et al., 2018), transcripts lack such multimodal depth. Nevertheless, child-directed speech from CHILDES provides an important window into the child language environment and is a rich target for computational analysis.

## Word learning within structured semantic environments

While acquiring language, children do not learn words at random. Words that occur more frequently and appear in multiple contexts tend to be learned earlier, but mere exposure is not the only driving factor. Advancements in graph theory have allowed researchers to examine word learning using network analysis of semantic similarity structure (Beckage et al., 2011; Dubossarsky et al., 2017; Engelthaler & Hills, 2017; Hills et al., 2009, 2010; Jimenez & Hills, 2017; Steyvers & Tenenbaum, 2005). Within a semantic network, words are represented as nodes, and words that are semantically related are connected by links. Semantic networks, like the Internet and most biological and social networks, exhibit a *small-world structure* (Barabási, 2016; Beckage et al., 2011; Cancho & Solé, 2001; Salathé et al., 2010): a small number of nodes have a high degree (many links with other nodes), while most have a low degree (few links with other nodes; Humphries & Gurney, 2008; Watts & Strogatz, 1998). Most paths between nodes that require more than one link will tend to pass through one of the high-degree “hub” nodes.

**Fig. 1:**
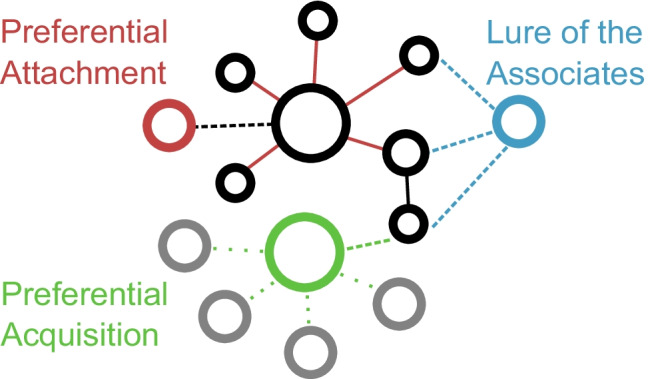
Depiction of network growth under three growth models. In our analyses, nodes correspond to words. The full set of nodes correspond to all the words in the semantic environment, and the edges (both solid and dotted) reflect all the associations that exist in the full network (i.e., in the environment). Black nodes represent the words that are currently known (i.e., the vocabulary at month m). Colored nodes indicate the word that is most likely to enter the growth model by month m + 1 according to each growth model: preferential attachment (red), lure of the associates (blue), or preferential acquisition (green). Grey nodes indicate other words in the environment. Solid edges connect words in the current vocabulary; dashed edges indicate associations between the three candidate words and the words in the current vocabulary; dotted edges indicate assocations between unknown words and candidate words. Edges are colored to reflect their relevance to each growth model. Preferential attachment expects that the red node will be acquired because it is associated with a known word that has degree 5 with respect to other words in the current vocabulary, while the green node is associated with just one known word with degree 1 (with respect to known words) and the blue node is associated with 3 nodes with a net degree of 4 (with respect to known words). Lure of the associates expects that the blue node will be acquired because it is associated with many known words, while the red and green nodes are associated with only one known word each. Preferential acquisition expects that the green node will be acquired because it is associated with a large number of known and unknown words: it has degree 5 with respect to the full environment, while the red nodes had degree 1 and the blue degree 3. Note that for this example edges are treated as undirected for simplicity. Subsequent analyses are applied to directed networks, and growth models are defined in text with respect to indegree and outdegree as appropriate

How network structure informs growth in semantic networks is unresolved. A major point of distinction between hypotheses is whether it is most important to consider the semantic structure within a child’s current vocabulary or the semantic structure of the environment the child engages with, including words they do not currently know. If the emphasis is on the structure of the current vocabulary, new words may be learned via *preferential attachment* (Steyvers & Tenenbaum, 2005): the next word that the child will learn is more likely to be associated with a known word that is central within the current vocabulary than with a known word that has few associations within the vocabulary. Conversely, if the emphasis is on the structure of the environment, new words may be learned by *preferential acquisition* (Hills et al., 2009): the next learned word is more likely to be associated with many other words in the environment, regardless of what words are currently known. A third alternative, dubbed the *lure of the associates,* would predict that the next learned word is more likely to be associated with many known words, regardless of the semantic structure within the current known vocabulary or among the words in the environment remaining to be learned. Figure 1 depicts each of these growth models with a simplified lexical network.

Determining which of these growth hypotheses is most in line with the typical development of lexical knowledge (vis-à-vis AoA norms) is relevant to theories of learning and cognition. Growth via the lure of the associates is consistent with a learning process that is insensitive to the aggregate structure of the environment as a whole and instead is sensitive only to how often words in the environment tend to co-occur with familiar words and concepts. Growth via preferential attachment is consistent with a learning process that is leveraging internal conceptual structure to learn from the environment and assimilate new knowledge. While the notion of internal structure here is too vague to truly map to a single theoretical perspective, it is particularly consistent with a constructionist “child-as-theorist” take on learning (Gopnik et al., 1999; Gopnik & Meltzoff, 1998; Waxman & Gelman, 2009). Conversely, growth via preferential acquisition is consistent with a distributional “child-as-analyst” take on learning (McClelland et al., 2010; Saffran et al., 1996; Wojcik & Saffran, 2013). However, although these theoretical connections can be made, the alignment between network growth profiles and the theoretical perspectives is not transparent, and the theoretical perspectives themselves are not mutually exclusive (Huebner & Willits, 2018; Waxman & Gelman, 2009).

## Current study

We have identified two important challenges for the study of child language acquisition, namely that (1) it is influenced by the semantic structure in the child’s environment and (2) insight into the child’s semantic environment is hard to obtain. We also note that child-directed speech differs in many critical ways from general language use and that child-directed speech is the most heavily weighted input for early language development. In other words, adults produce the language that constructs the child language environment through a context-sensitive adaptation of their usual language.

The current study therefore considers whether a child-oriented context can be induced for adult participants without involving any children. We adapted the instructions for the word association task to direct participants to respond as if playing an association game with a toddler (i.e., a 2- to 3-year-old). We first assess whether the distribution of responses differs when compared to a task administered with conventional instructions but involving the same cue words drawn from the McArthur–Bates Communicative Development Inventory (CDI, Fenson et al., 2007). We collected our own control data, rather than drawing from the USF or SWOW free association databases, to ensure maximal coverage of words on the CDI and to make responses from the two conditions as comparable as possible. We then assess the semantic structure implied by the child-oriented and unconstrained response profiles for each word, and whether they differ. Finally, we consider whether this variation on the word association task provides unique insight into how young vocabularies grow by comparing network growth trajectories based on association networks derived from child-oriented and adult-oriented norms.

## Methods

### Word association participants

Participants were recruited using Amazon Mechanical Turk and Prolific, which are both online crowd-sourcing platforms. Before data collection began, approval was granted by Louisiana State University’s Institutional Review Board. Eligible individuals were native English speakers, 18 years or older, and currently living in the USA. These criteria could be partially enforced by the platforms themselves, which require age, language, and location information to register. The native English requirement was announced in the title of the job ad, and our task began by asking people to confirm that their native language is English. Non-native English speakers were not allowed to proceed with the experiment. Participants were required to report their age along with other demographic information after the primary task. The tasks were only available to participants residing in the United States.

Participants took 13 minutes and 20 seconds on average to complete the study and were compensated $3 ($13.50/hour average rate). Individuals could participate multiple times, each time completing a different word list or experimental condition but were prevented from responding to the same words in the same condition more than once. We recruited 1864 individuals to complete 4101 experimental sessions divided among two task conditions, which we will refer to as *adult-oriented* (*n*_*adult*_ = 2047) and *child-oriented* (*n*_*child*_ = 2054) as described below. The participants varied widely in age, levels of income, academic achievement, and race. Participants were 48.5% male, and males contributed 55.4% of the responses (they were more likely to participate more than once). The participants predominantly reported being White/Caucasian (71.9%) and non-Hispanic (92.5%). All adult-oriented respondents were recruited using MTurk (620 participants completing 2047 sessions), while child-oriented respondents were recruited using both platforms (MTurk: 311 participants completing 993 sessions; Prolific: 933 participants completing 1061 sessions). Responses were pooled across recruitment platforms—although there are interesting differences in demographics and engagement with the platform between the MTurk and Prolific communities, they did not manifest in significant differences in association responses across platforms.

Data were excluded following the criteria enforced in the Small World of Words study (De Deyne et al., 2019). Participants who provided >30% multi-word responses, >40% non-English responses, or >20% nonunique responses were removed from the dataset. Participants also were removed if they provided off-task responses to an attention-monitoring question that appeared within the task. We also visually inspected participant responses for obviously off-task responses that might evade these criteria (such as someone writing out an English sentence one word at a time). Behavior of this kind was rare, and such sessions were excluded from the numbers reported above.

### Word association tasks

On each trial, a single word was presented at the top of the screen, above three vertically arranged text boxes. Participants were instructed: “Type the first word that comes to mind when reading this word. Press TAB to type a second and third word that comes to mind. Click the ‘Next’ button to proceed to the next trial.” Additionally, they were explicitly instructed to respond only to the cue word and to not “chain” responses (i.e., provide an associate of a previously provided response) and to provide single-word responses without abbreviation. Emphasis was put on reporting the first words that came to mind, rather than seeking a “best” answer. Additionally, one question appeared within the task that monitored for participant engagement (“List the colors of the American flag.”). Participants who failed to respond with some combination of “red”, “white”, and “blue” were excluded. The task was implemented and presented to participants online using Qualtrics software (February–July 2019. Copyright © 2020 Qualtrics. Qualtrics and all other Qualtrics product or service names are registered trademarks or trademarks of Qualtrics, Provo, UT, USA. https://www.qualtrics.com.)

The child-oriented condition was created by providing a cover story: “Imagine you are playing a game with a toddler (a 2- to 3-year-old child). In this game you draw a card with a word on it and say the first 3 words that come to mind. Over the course of the game, you will draw many cards and expose the toddler to many related words. Each of the following screens is like a card draw. You should type the first three related words you would say to the toddler. After filling in the first box, you can press TAB to move to the next box. Press the ‘Next’ button to proceed to the next trial.” To further orient participants, the instructions were presented alongside an image of a male toddler, so those who are not around children often might have a better intuition about the age we were targeting. This child’s face was present on the screen throughout the experiment as a reminder of the instructions. The same image was used for all participants. As with the unconstrained version of the task, the child-oriented condition contained an item to monitor for participant engagement in the task (“List the colors of the American flag.”).

### Word association cues

Cues were selected from the McArthur–Bates Communicative Development Inventory (CDI) Words and Sentences form (Fenson et al., 2007), which is intended for toddlers between 16 and 30 months of age and consists of 680 items classified as words. From these, we excluded three items that refer to idiosyncratic proper nouns (“babysitter’s name”, “child’s own name”, “pet’s name”) and four short phrases (“give me five!”, “gonna get you!”, “so big!”, “this little piggy”). Other short phrases, such as “a lot”, “all gone”, and “next to”, were retained. To target the intended meaning on the CDI, we included disambiguating cues (e.g., “chicken [food]”, “chicken [animal]”).

The 672 cue words were split randomly into 20 lists that were comprised of 30–35 words with no coherent theme. Within each group, the words were sequenced so that neighbors were not semantically similar. These steps were taken to eliminate dependence among cues. For example, “PIG” followed by “SHEEP” may establish a “farm” context, while “BED” followed by “SHEEP” may establish a “sleep” context. A participant would be presented the sequence in forward or reverse order—this was done in case responses to words earlier in the list received systematically different responses than those later in the list (e.g., due to fatigue). Thus, at least 50 participants were randomly distributed to each condition (unconstrained or child-oriented), cue list (1–20), and sequence order (forward or reverse). Responses were collapsed across forward- and reverse-ordered lists.

Response data were manually screened for nonsensical responses as the data were being collected. The retained participants always provided three reasonable responses to each cue. At the end of data collection, each cue in the adult-oriented condition had three responses from at least 100 participants, and each cue in the child-oriented condition received three responses from at least 97 participants (402 cues had 100 participants, 137 had 99, 101 had 98, and 32 had 97). Responses were then cleaned by forcing all responses to lower case, removing extraneous white space, and correcting cases where a letter was repeated more than twice consecutively (“loook” ➔ “look”). Following this, and after observing that responses did not significantly differ by sequence order, data were aggregated. In cases where more than 100 participants were recruited, only the first 100 were retained for analysis.

### Child-directed natural language corpus

Language transcripts from the North American section of the CHILDES database were filtered and selected according to the child’s age. Transcripts with children between the ages of 3 and 60 months were selected when the role of the speaker was “Adult”, “Father”, “Mother”, “Aunt”, “Uncle”, “Grandmother”, “Grandfather”, “Teacher”, “Babysitter”, “Nurse”, “Doctor”, “Clinician”, or “Therapist” (all adult caretaker roles). Given that transcripts have been contributed from various labs, slight variations in coding appear; therefore, we carefully assessed the transcripts and codes to appropriately resolve inconsistencies. During transcript processing, we tokenized words (split on spaces) and regularized spellings so that differently spelled words were converted to the same form. Nouns and verbs also were morphologically parsed (e.g., splitting plural and possessive markers, splitting past-tense markers). An automatic text stemming program (**textstem** for R) was used to generate a dictionary of words and lemmas (Rinker, 2018) that was then manually reviewed and corrected (e.g., preventing “disgusting” from being stemmed to “gust”). Next, common phrases like “thank you”, “all gone”, and “go potty” were tokenized (i.e., reduced from two adjacent tokens to a single token). Common variants were regularized (“ya” to “you”); nonwords, proper nouns, and instances of babbling or signing were replaced with special tokens (e.g., “__childinvented__”, “__name__”, “__babble__”). Following these cleaning procedures, we were left with 4.5 million tokens.

### Estimating age of acquisition from language production norms

For the purposes of modeling lexical growth, we estimated age of acquisition (AoA) from production norms established by a sample of 5520 American English-speaking 16- to 30-month-old children whose parents completed the CDI vocabulary checklist (Fenson et al., 2007). These data were contributed by researchers around the USA and made publicly available via Stanford Word Bank (Frank et al., 2017). An AoA was estimated for each of the 672 cue words as follows. For each of the 5520 children, the child’s age is documented and whether they produce each word at the time of assessment. For each cue word, a logistic model can be fit that can produce a probability of production for each age. Based on this model, one can estimate the age at which the probability of production is 0.5, and this is taken as the AoA for that word (Goodman et al., 2008). Fifty-one words on the CDI were not produced by at least 50% of children by 30 months, which prevented AoA from being estimated for these words.

### Network estimation

The network structure of the semantic environment can be estimated from word association data by treating each cue word as a node in a network and drawing directed connections between cues: if cue_A_ is provided as a response to cue_B_, then cue_B_ → cue_A_. Thus, asymmetric adjacency matrices were constructed using the cue and response data that we collected in each of our (two) word association tasks. To facilitate analysis, we excluded the 12 words that repeat across categories on the CDI (24 items total) and 51 words for which age of acquisition (AoA) could not be estimated (one of which was a repeat); we also excluded these words from all subsequent networks. Appendix A identifies the CDI words that were excluded from the network analyses. Thus, each network consists of 598 nodes, corresponding to 598 CDI items that children between 16 and 30 months would be expected to know.

An additional asymmetric adjacency matrix was derived from word co-occurrence statistics within the CHILDES child-directed speech transcripts. To obtain directed connections, we tracked which words followed other words within five-token forward-looking moving windows, accruing evidence for a connection from the first word in the window to each of the four that follow it. The co-occurrence of the first token in the window with each of the subsequent tokens was tabulated, forming an asymmetric type-by-type association matrix (Hills et al., 2010; Jimenez & Hills, 2017). The network was then filtered to retain only nodes corresponding to the 598 CDI items described above.

### Node indegree and lexical growth values

The development of a vocabulary can be understood as the sequential acquisition of words from a set of possible words. In this work, the relationships among the possible words are expressed as a network defined to reflect semantic relationships and may be derived from word associations or transcripts of child-directed speech. Statistics can be computed for each word to emphasize different aspects of their position within the network. The *centrality* of a network node can be measured in many ways. One simple and common metric is the number of connections that terminate on a node. This is called the node’s *indegree*. Prior work indicates that a word’s indegree is predictive of lexical and semantic behavior (De Deyne et al., 2013) and has been used in previous work modeling lexical growth (Hills et al., 2009; Stella et al., 2017).

Many well-studied networks, like the Internet and biological systems, grow by preferentially attaching new nodes to previously acquired nodes with high indegree relative to other acquired nodes (Steyvers & Tenenbaum, 2005). Prior work indicates that early language learners may preferentially acquire words that are central to their semantic environment overall. This suggests that lexical networks grow differently than other types of networks. The critical contrast is whether network growth is driven by the structure of the environment (preferential acquisition), the structure among the subset of the environment that is already acquired (preferential attachment), or the child’s existent lexical knowledge (lure of the associates).

For each month from 16 to 30, we categorized whether each CDI word was known or unknown using the AoA data derived from WordBank child data. Then, starting at month 16, the youngest for which we have CDI data on the Words and Sentences form, we considered the subset of the full network consisting only of nodes corresponding to words known at 16 months. Then we computed “growth values” relative to this 16-month subnetwork according to each model of growth (preferential attachment, preferential acquisition, lure of the associates). This process was repeated for each month, each time calculating growth values for a different set of unknown words relative to a different subnetwork reflecting typical children of increasing age. The growth values computed based on the words known at 16 months are expected to be largest for words that will be learned by the next month (i.e., with an AoA of 17 months). Under preferential attachment, the growth value is equal to the average of the indegree of all currently known words to which the new word would attach. Under lure of the associates, the growth value is equal to the sum of the indegree of the unknown words (i.e., the sum of the known words that link to the unknown word). In contrast, under preferential acquisition, the growth value of an unknown word is simply its own indegree in the context of all of the CDI words for which we had AoA values (regardless of what words are currently known by the average child).

At each subsequent month, the set of known words grows (according to AoA), and the growth values associated with preferential attachment and lure of the associates are recomputed for each unknown word—growth values associated with preferential acquisition are independent of what words are currently known. Once growth values are known for months 16–29 (the CDI does not assess children older than 30 months), the values at each month are standardized to have mean 0 and standard deviation 1.

The datasets and scripts that were used in the current study are available in the OSF repository https://osf.io/3pmcw.

## Results

### Child-oriented associations differ from unconstrained adult-oriented associations

Our first research aim was to determine whether the child- and adult-oriented association tasks elicit different responses. We predicted that child-oriented responses would consist of higher-frequency words that are shorter and acquired earlier in life (lower AoA). These predictions were tested in a series of within-cue factorial ANOVAs. Each ANOVA was defined with condition (adult-oriented vs. child-oriented) and response order (first, second, or third response) as independent variables, and applied to the average values for each cue, condition, and ordinal response. Separate models were conducted for the following dependent variables: SUBTLEX word frequency (Brysbaert & New, 2009), number of letters, number of phonemes, number of syllables, and age of acquisition (Kuperman et al., 2012), and contextual diversity (Brysbaert & New, 2009).

**Fig. 2:**
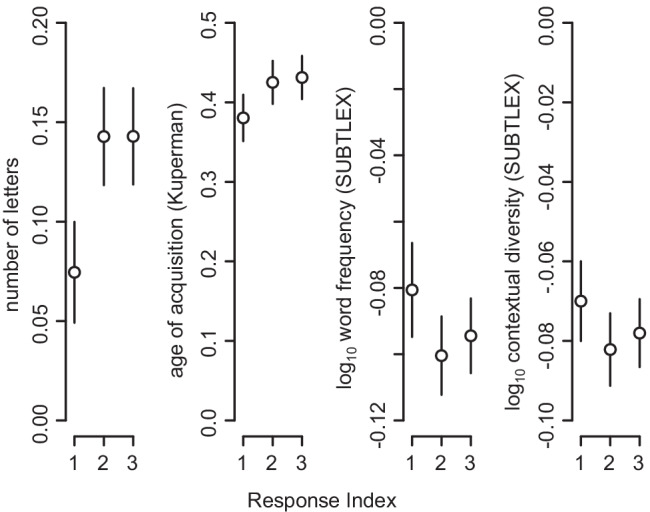
Mean of differences between adult- and child-oriented responses within-cues for each response index. Positive values indicate that the value is larger in the adult-oriented condition. For number of letters, age of acquisition, and word frequency, the interaction between condition and response order is significant. Errorbars reflect 95% confidence intervals

All ANOVA results are presented in Table 1, and descriptive data for each dependent variable by condition and response order are presented in Table 2. Main effects of condition and response order were observed for all six dependent variables. For all dependent variables except contextual diversity, condition and response order significantly interacted. Every significant interaction indicates the same moderating effect: responses in the adult-oriented condition became increasingly “complex” (longer, lower frequency, higher AoA) with response order, while this drift toward complexity was attenuated in the child-oriented condition. That is, while differences by condition are observed for each of the three response positions, the differences are larger for the second and third response than they are for the first. Mean paired differences, reflecting paired *t*-tests evaluating the simple effects of condition at each level of response order, are plotted in Fig. 2 with 95% confidence intervals. All tests have 597 degrees of freedom and are significant (*p* < .001). We omit plots for syllables and phonemes because they are very similar to the plot for number of letters and these dependent variables are highly correlated. Simple effects of response order at each level of condition are also significant for all five dependent variables for which condition and response order interact (*p* < .001). The condition effect was predicted given that words that are used frequently and in a variety of contexts are learned more easily than words that have more restricted use (Hills et al., 2010; Johns et al., 2016).

**Table 1:** Response statistics: repeated-measures ANOVAs

Statistic	Predictor	df_1_	df_2_	*ϵ*	F	*p*	${\eta}_G^2$
Letters	*Condition*	1	597		165.02	<.001	0.01
	*Response*	1.30	776.13	0.65	174.88	<.001	0.04
	*Cond × Resp*	1.96	1170.79	0.98	14.67	<.001	0.00
Phonemes	*Condition*	1	597		167.90	<.001	0.01
	*Response*	1.32	787.19	0.66	182.25	<.001	0.05
	*Cond × Resp*	1.98	1182.45	0.99	13.16	<.001	0.00
Syllables	*Condition*	1	597		116.98	<.001	0.01
	*Response*	1.29	772.67	0.65	98.74	<.001	0.03
	*Cond × Resp*	1.97	1178.51	0.99	16.50	<.001	0.00
Age of acquisition	*Condition*	1	597		1428.18	<.001	0.12
	*Response*	1.40	834.27	0.70	390.35	<.001	0.08
	*Cond × Resp*	1.97	1176.49	0.99	6.29	.002	0.00
Frequency	*Condition*	1	597		353.75	<.001	0.01
	*Response*	1.32	788.24	0.66	215.94	<.001	0.02
	*Cond × Resp*	1.95	1162.20	0.97	4.15	.017	0.00
Contextual diversity							
	*Condition*	1	597		468.59	<.001	0.02
	*Response*	1.37	816.92	0.68	207.48	<.001	0.02
	*Cond × Resp*	1.97	1176.24	0.99	2.64	.072	0.00

*Note.* df_1_ indicates degrees of freedom numerator. df_2_ indicates degrees of freedom denominator. Epsilon (*ϵ*) indicates Greenhouse–Geisser multiplier for degrees of freedom; *p*-values and degrees of freedom in the table incorporate this correction. ${\eta}_G^2$ indicates generalized eta-squared. Condition levels: adult-oriented, child-oriented. Response levels: 1, 2, 3

**Table 2:** Response statistics: means and standard deviations

	Condition	1	2	3
Letters	*Adult*	4.845 (0.64)	5.09 (0.49)	5.15 (0.44)
	*Child*	4.77 (0.69)	4.95 (0.49)	5.01 (0.43)
Syllables	*Adult*	1.340 (0.24)	1.48 (0.19)	1.49 (0.17)
	*Child*	1.37 (0.27)	1.42 (0.20)	1.44 (0.17)
Phonemes	*Adult*	3.96 (0.55)	4.18 (0.44)	4.23 (0.39)
	*Child*	3.89 (0.60)	4.05 (0.43)	4.10 (0.38)
Age of Acquisition	*Adult*	4.88 (0.67)	5.17 (0.57)	5.30 (0.52)
	*Child*	4.50 (0.62)	4.74 (0.50)	4.87 (0.45)
Frequency	*Adult*	3.51 (0.51)	3.39 (0.41)	3.36 (0.38)
	*Child*	3.59 (0.53)	3.49 (0.44)	3.46 (0.41)
Contextual Diversity	*Adult*	3.13 (0.32)	3.05 (0.27)	3.03 (0.25)
	*Child*	3.20 (0.32)	3.13 (0.28)	3.11 (0.26)

*Note.* Columns labeled 1, 2, 3 correspond to the first, second, and third response to each cue. Standard deviations are shown in parentheses

It is possible that the effect of the child-oriented task manipulation is moderated by the age of acquisition (AoA) of the cue. We consider this in a supplemental analysis reported in detail in Appendix B. In short, there is a positive linear relationship between the AoA of the cue and the AoA of the responses, but the condition effect is remarkably stable over cues.

### Child- and adult-oriented associations express different similarity structure among cues

Our second research aim was to determine whether the two word association tasks would yield distinct semantic similarity structures. It is possible that the responses provided in the child-oriented condition differ significantly without implying different semantic relationships among the cue words. For instance, if in response to the word STAR people tend to respond with LUMINOUS in the unconstrained condition and BRIGHT in the child-oriented condition, this conveys similar information about STAR and implies similar relationships to other cues.

To test this, we first cross-tabulated cues and responses separately for each condition. This yielded two tables, with a column for each of the 672 cues and a row for each unique response generated in the respective condition. This is not a network representation of the word association data; instead, we are quantifying the similarity between each cue based on how many responses they share. To ensure that our analysis was not dominated by frequency effects, we replaced all nonzero values in these tables with ones before computing Pearson’s *r* for each pair of columns. This yields two 672 × 672 matrixes of correlation coefficients, one for each condition. Then, following convention for representational similarity analysis (Nili et al., 2014), we computed the Spearman rank correlation between the lower triangle of the two matrices (excluding the diagonal). This correlation is an estimate of the matrices’ “representational similarity” and will be high if the relationships among cues are similar across task conditions and will be low if they differ.

**Fig. 3:**
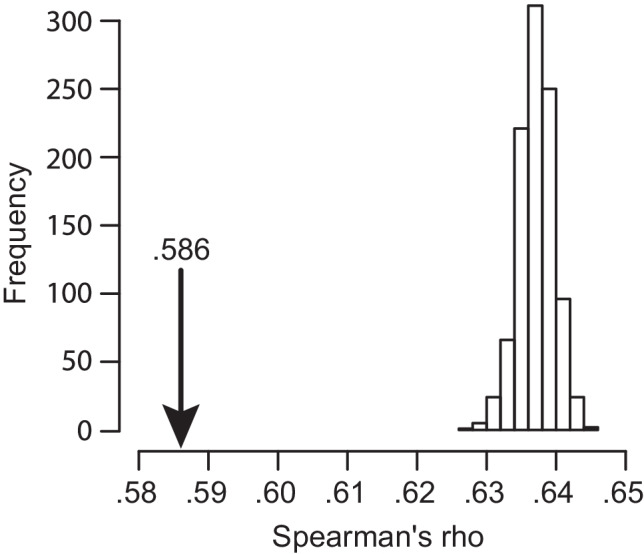
Representational similarity between adult- and child-oriented semantic environment estimates (arrow). Histogram reflects the empirical null distribution, simulated by combining and randomly splitting the responses to each cue and repeating the representational similarity analysis.

To assess whether the representational similarity between conditions is lower than we would expect based on an arbitrary split of our dataset, we combined data from both conditions by cue, and split the responses to each cue in half randomly 1000 times. For each of these splits, we repeated the analysis above, resulting in 1000 representational similarity values relating the content of the halves. While the representational similarity between our child-oriented and adult-oriented conditions was *r* = .586, random splits were associated with average $\overline{r}=.637$ (*σ*_*r*_ = .002). No random split had a lower representational similarity than that associated with the true split between conditions, indicating that the responses in the child-oriented condition imply different relational structure among the cue words (see Fig. 3).

We further examined the semantic structure of the networks derived from the two word association tasks by inspecting the associative paths among all 598 cues as networks. The procedure for constructing associative networks is described in the Methods section. We calculated the average shortest path length, average of local transitivity (i.e., clustering coefficient), and small-world index (SWI; Neal, 2017). As can be seen in Table 3, the semantic network based on the child-oriented word association data are more clustered and have an overall higher SWI relative to the semantic network derived from the adult-oriented word association data. Simulating null distributions for the difference on each network statistic using the same method described above indicates that the difference between the clustering coefficients in each condition is larger than would be expected if all responses were sampled from the same condition (*p* < .001). The networks do not reliably differ on path length or SWI. Our network comparisons suggest that the child-oriented semantic environment differs both in structure and semantic content.

**Table 3:** Semantic network structure characteristics relative to word association task condition

	Average shortest path length	Average of local transitivity (clustering coefficient)	Small-world index (Neal, 2017)
Adult	2.504	2.255	0.300
Child	2.584	2.465	0.450
Difference	0.08 [−2.76, 3.07]	0.21 [0.20, 0.22]	0.15 [−0.43, 0.75]

*Note.* Small-world index is computed as $\frac{L-{L}_l}{L_r-{L}_l}\times \frac{C-{C}_r}{C_l-{C}_r}$, where *L* refers to the average shortest path length, *C* refers to the clustering coefficient, and subscripts *r* and *l* refer to randomized and “latticized” versions of the network being described. It ranges between 0 and 1, where 1 is the most ideal small-world network. Following each difference score is a 95% confidence interval derived from simulating the null distribution as described in the text

### Word associations and transcripts of child-directed speech provide different perspectives on the semantic environment

The modified word association task is one of many ways one might attempt to estimate the structure of the child’s semantic environment. An obvious alternative approach used in previous work considers the distributional statistics of child-directed speech, facilitated by the freely available CHILDES child language database (MacWhinney, 2000). Though both methods will yield an estimate of semantic structure among a set of cues, they provide different perspectives on the semantic environment. For example, one would expect word associations to reveal more taxonomic (i.e., categorical) structure than co-occurrence in natural language.

To test this, we grouped the words on the CDI by the 22 categorical headings on the Words and Sentences form. Each pair of words can then be labeled as within-category or between-categories. We then computed the shortest distance between each pair of nodes within the child-oriented, adult-oriented, and CHILDES networks. Finally, we computed the average distances for within-category and between-category pairs for each network and report a ratio of within distances to between distances. Networks that reflect more taxonomic structure will have lower ratios (shorter distances within groups than between groups). A difference of ratios between networks quantifies structural differences between those networks. Two-tailed 95% confidence intervals were constructed around these differences via nonparametric bias-corrected and accelerated (BCa) bootstrap using the boot package in R (1000 replicates).

The taxonomic ratios for the adult-oriented (0.888, SE = 0.002) and child-oriented (0.875, SE = 0.002) association networks both differed from the CHILDES (0.958, SE = 0.003) association network. The adult- and child-oriented networks differed minutely from each other—5.8 times less than the difference between CHILDES and the mean of the adult and child ratios. This is consistent with a taxonomic bias in the networks based on word associations. These results are summarized in Table 4.

**Table 4:** Differences among taxonomic ratios

		Difference	SE	95% CI (BCa)
CHILDES	Child	0.083	0.004	[0.075, 0.090]
CHILDES	Adult	0.070	0.004	[0.062, 0.077]
Child	Adult	0.013	0.003	[0.007, 0.018]

*Note.* Standard error and confidence intervals are estimated based on 1000 bootstrap replicates. The estimated bias was <0.001 for all confidence intervals. *SE* standard error, *CI* confidence interval, *BCa* bias-corrected and accelerated bootstrap

### Semantic network structure predicts normative lexical growth

Our third research aim was to determine whether the child-oriented and adult-oriented word associations differentially predict vocabulary growth. We begin by constructing two networks, both with nodes corresponding to words with an estimated AoA of 16, but with directed edges inserted with respect to either the child-oriented or adult-oriented associations. Then each unknown word is evaluated with the three network-growth models (preferential attachment, preferential acquisition, and lure of the associates). Each model assigns a “growth value” that is proportional to the strength of the expectation that the unknown word will be the next node added to the network. The set of growth values associated with each network-growth model are then z-scored before selecting the values assigned to words that are expected to be learned next—in this case, those with an AoA of 17. This process is repeated iteratively for child- and adult-oriented networks representing vocabularies from 16 to 28 months. After iterating, this yields 547 values per growth model and condition, which is 598 less the 18 words in the initial 16-month network and the 33 words that are learned in month 30.

**Fig. 4:**
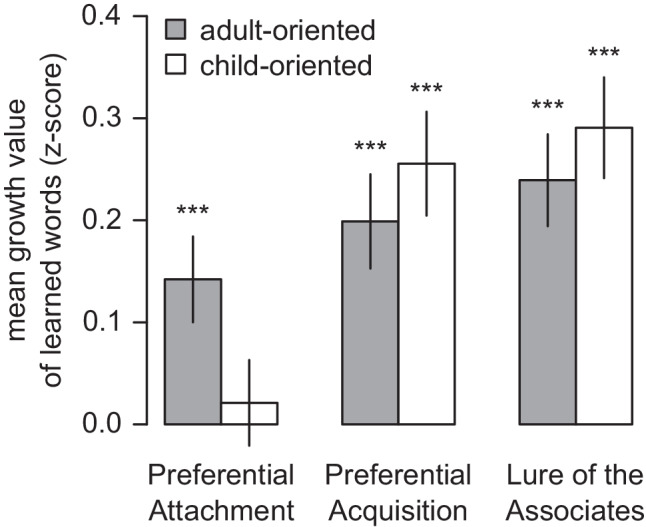
Mean growth values under each growth model, z-scored relative to unknown words at the month of acquisition. Error bars indicate standard error of the mean. Stars indicate result of t-test against zero. * *p* < .05, ** *p* < .01, *** *p* < .001

Mean standardized growth values for each cell in this factorial design are shown in Fig. 4. Except for the preferential attachment model applied to the child-oriented association network, one-sample *t*-tests indicate that the means are unlikely to have arisen from a null distribution centered on zero (*t*(545) ≥ 3.57, *p* < .001). Note that zero would be the expected standardized value if the growth models were not predictive of vocabulary growth. This confirms that the growth models are informative about what words will be learned next and echoes prior work indicating that growth by preferential attachment is the least consistent with typical patterns of language acquisition. However, the degree to which this is possible may differ depending on whether associations are defined according to the child- and adult-oriented network; we examine this in the following section.

**Child-oriented associations predict word learning more accurately**

We further examined our third research aim by testing the contribution of each growth model and network structure played in explaining variance in growth values. The variance introduced by manipulating the growth model (preferential attachment vs. preferential acquisition vs. lure of the associates) and the network structure (child- vs. adult-oriented) was modeled within cues as a 3-by-2 repeated measures ANOVA. There was a main effect of model and a significant model by network interaction (Table 5).

**Table 5:** Within-cues ANOVA of growth values

Predictor	df_1_	df_2_	*ϵ*	F	*p*	${\eta}_G^2$
*Network*	1.00	546.00		0.01	.912	0.00
*Model*	1.14	620.75	0.57	5.80	.013	0.00
*Model × Net*	1.13	615.73	0.56	12.74	<.001	0.00

*Note.* df_num_ indicates degrees of freedom numerator. df_den_ indicates degrees of freedom denominator. Epsilon (*ϵ*) indicates Greenhouse–Geisser multiplier for degrees of freedom; *p*-values and degrees of freedom in the table incorporate this correction. ${n}_G^2$ indicates generalized eta-squared

Given the significant model by network interaction, we conducted simple effects comparisons between networks (paired *t*-tests) for each model. The child-oriented network yielded higher standardized growth values (i.e., better predictions about language growth) than the adult-oriented network when applying the preferential acquisition (*t*(546) =  − 2.58, *CI* = [−0.101, −0.014] ,*p* = 0.010, *d* = 0.110) and lure of the associates models (*t*(546) =  − 2.22, *CI* = [−0.102, −0.006], *p* = 0.027, *d* = 0.095), while the opposite effect was obtained with the preferential attachment model (*t*(546) = 2.73, *CI* = [0.033, 0.204], *p* = 0.006, *d* = 0.117).

Repeated-measures ANOVAs exploring the simple effects of the growth model manipulation for each network separately indicate that the growth models perform similarly when applied to the adult-oriented network but differ when applied to the child-oriented network (Table 6). This effect is driven by the poor performance of the preferential attachment model on the child-oriented network (Fig. 4); preferential acquisition and lure of the associates did not differ significantly (paired *t*(546) = 1.86, *CI* = [−0.002, 0.077], *p* = .063).

**Table 6:** Simple effects by network

Network	Predictor	df_1_	df_2_	*ϵ*	F	*p*	${\eta}_G^2$
Adult	Model	1.13	617.32	0.57	1.07	.310	0.00
Child	Model	1.14	623.59	0.57	12.31	<.001	0.01

*Note.* df_1_ indicates degrees of freedom numerator. df_2_ indicates degrees of freedom denominator. Epsilon (*ϵ*) indicates Greenhouse–Geisser multiplier for degrees of freedom; *p*-values, and degrees of freedom in the table incorporate this correction. ${n}_G^2$ indicates generalized eta-squared

### Network structure improves predictions of word learning beyond psycholinguistic factors

The preceding analysis of standardized growth values indicated that the three growth models, particularly preferential acquisition and lure of the associates, are predictive of the typical progression of word learning. We now adopt a model comparison approach that can help isolate the informativeness of an associative network structure in predicting vocabulary growth relative to more basic psycholinguistic factors and other networks (Hills et al., 2009). Relative to the expected vocabulary at each month 16–29, the probability of learning each unknown word is estimated based on a ratio of strengths:

$${p}_i=\frac{e^{\beta {x}_i}}{\sum_j{e}^{\beta {x}_j}}$$

In this equation, *x*_*i*_ and *x*_*j*_ represents column vectors of word-level variables, including psycholinguistic variables and potentially the value associated with one or more network growth models. The subscript *i* denotes the currently unknown word for which the probability of learning is being estimated, and the subscript *j* iterates over the set of unknown words at the month word *i* is expected to be learned. These vectors are matrix multiplied with the row vector *β*, which is a constant set of weights applied to scale and sum the variables in *x*. Solving the equation yields a single probability, *p*_*i*_. The log-likelihood of the model is obtained by taking the sum of the log transformed probabilities for all learned words:

$$\log \theta \left(\beta \right)=\sum \log {p}_i$$

The vector *β* is optimized for a given set of variables using the stats::optim function in R (R Core Team, 2020). Nested models can be compared using a likelihood-ratio test—the difference of log likelihoods follows a *χ*^2^ distribution (*θ*_0_ denotes the likelihood of the restricted model, and *θ*_1_ denotes the likelihood of the full model in the nested pair):

$$-2\left(\log {\theta}_1-\log {\theta}_0\right)\sim {\chi}^2$$

Kover and Ellis Weismer (2014) demonstrated that children with typical and delayed language development tend to learn short words with high phonological neighborhood densities at early points in lexical development. Additionally, Schneider et al. (2015) found that young children begin their word-learning journey by learning high-frequency words with low phonological complexity. Thus, we fit a baseline model including psycholinguistic variables that are known to influence word learning but are unrelated to the associative semantic structure of the language: number of phonemes, word frequency (calculated from the CHILDES corpus; Bååth, 2010), phonotactic probability, and phonological neighborhood density (estimated using the phonological neighborhood calculator; Vitevitch & Luce, 2004, 2016). We then fit additional models incorporating growth values derived from our child- and adult-oriented word association networks. In addition, to compare the network structure obtained from our word association tasks to structure learned from a child-directed natural language corpus, we generated growth values for a network derived from word co-occurrences in transcripts of child-directed speech publicly available through CHILDES as described in the methods (McWhinney, 2000).

The correlations among predictor variables are reported at the OSF repository for this paper. Preferential attachment growth values are strongly correlated across all networks (*r* ≈ .9); preferential acquisition and lure of the associate growth values are strongly correlated between the two networks based on word association data (*r* ≈ .9) and far less correlated with values derived from the CHILDES network (*r* ≈ .45). Growth values derived from the CHILDES network are more correlated with the psycholinguistic baseline variables (${R}_{CHILDES}^2=.457$) than growth values derived from the other two networks (${R}_{adult}^2=.137$, ${R}_{child}^2=.101$). When constructing models that include multiple growth values from one network, or when constructing models that include the same growth value derived from different networks, collinearity is an issue. Collinearity increases the standard error for all model parameters and reduces the power of statistical tests. When including variables that are collinear, the explained variance that is unique to each independent variable is reduced, which may decrease the number of variables that significantly predict a dependent variable (Fox & Weisberg, 2011). As will be seen below, despite the collinearity that exists in our data, comparisons of nested models reveal that certain network variables explain vocabulary growth values better than others.

**Fig. 5:**
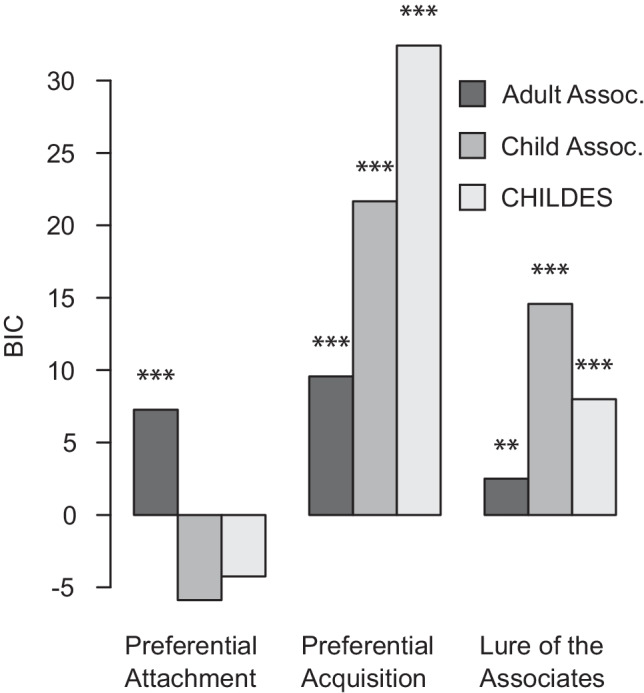
Nested model comparisons to psycho-linguistic baseline model. * <.05, ** <.01, *** <.001

Nested model comparisons against the psycholinguistic baseline model are summarized in Table 7; the Bayesian information criterion (BIC) for each model is plotted in Fig. 5. When applied to child-oriented sources, the preferential attachment growth model does not add predictive value over the psycholinguistic baseline. However, improvement is observed in every other case. Note that in our figures and tables, we compute BIC as:

$$\mathrm{BIC}=2\log \left({\theta}_1-{\theta}_0\right)-k\log n$$

where *k* refers to the difference in the number of variables between the full and restricted model, and *n* refers to the number of observations. Large positive BIC values indicate better model fit.

**Table 7:** Model comparisons against psycholinguistic baseline

Model	Network	df	log*θ*_0_	log*θ*_1_	*χ*^2^	*p*	*p* (FDR)	BIC
*Preferential attachment*	Adult	1	3099.078	3092.351	13.455	<.001	<.001	7.151
	Child	1	3099.078	3098.717	0.722	.396	.443	−5.583
	CHILDES	1	3099.078	3098.102	1.952	.162	.207	−4.352
*Preferential acquisition*	Adult	1	3099.078	3090.528	17.101	<.001	<.001	10.797
	Child	1	3099.078	3084.486	29.184	<.001	<.001	22.880
	CHILDES	1	3099.078	3073.576	51.005	<.001	<.001	44.701
*Lure of the associates*	Adult	1	3099.078	3093.165	11.827	.001	.001	5.523
	Child	1	3099.078	3086.280	25.597	<.001	<.001	19.292
	CHILDES	1	3099.078	3091.832	14.492	<.001	<.001	8.188

*Note.* The restricted model always consists of the psycholinguistic baseline variables, which is why log*θ*_0_ is the same for all comparisons. Each full model additionally includes the growth values based on each combination of growth model and network. All models predict probabilities for 580 words learned in months 16 through 29. FDR = false discovery rate

Controlling for variance that can be attributed to the psycholinguistic factors, the differences between adult- and child-oriented sources are more pronounced, and the preferential acquisition growth model appears to outperform the lure of associates (Fig. 5). This latter observation is confirmed by model comparisons reported in Table 8: a model that already includes the psycholinguistic variables and the lure of the associates growth values is improved by adding the preferential acquisition growth values, but the reverse is not true. The same pattern is observed regardless of how the network was defined (i.e., based on adult- or child-oriented word associations or CHILDES transcripts). Thus, the remainder of analyses will focus on the preferential acquisition growth model for simplicity—however, we note that the BIC associated with these significant differences is small (less than 2).

**Table 8:** Model comparisons between growth models

Network	M_0_	M_1_	df	log*θ*_0_	log*θ*_1_	*χ*^2^	*p*	*p* (FDR)	BIC
*Adult*	Acq.	LOA	1	3090.528	3090.523	0.010	.922	.993	–6.295
	LOA	Acq.	1	3093.165	3090.523	5.284	.022	.030	–1.021
*Child*	Acq.	LOA	1	3084.486	3083.434	2.105	.147	.196	–4.200
	LOA	Acq.	1	3086.280	3083.434	5.692	.017	.025	–0.613
*CHILDES*	Acq.	LOA	1	3073.576	3073.970	–0.789	1.000	1.000	–7.093
	LOA	Acq.	1	3091.832	3073.970	35.724	<.001	<.001	29.420

*Note.* All models include psycholinguistic variables. M_0_ refers to the growth model included in the restricted model, and M_1_ refers to the additional growth model added to construct the full model in the nested pair. All models predict probabilities for 580 words learned in months 16 through 29. *Acq*. preferential acquisition, *LOA* lure of the associates, *FDR* false discovery rate

### Word associations and child-directed speech provide complementary information about word learning

When considering the models against the psycholinguistic baseline, it appears that growth models based on transcripts of child-directed speech from CHILDES are more predictive than those based on the child-oriented word associations. While this would appear to undermine the utility of collecting child-oriented word associations to estimate the semantic environment of children, the variance in word learning may not be entirely redundant. To test this possibility, we conducted model comparisons between models that include two sets of growth values (e.g., from the adult-oriented and child-oriented networks) to models that only include one or the other. If the variance explained by the CHILDES growth model is a superset of the variance explained by the child-oriented associations, a full model that includes both should not perform better than a restricted model that only includes CHILDES. This is not what we observe. The model that includes both CHILDES and the child-oriented word association growth values performs substantially better than the model that only includes CHILDES growth values: *χ*^2^(1) = 26.565, *p* < 0.001, BIC = 20.201. This is consistent with the semantic structure available via the word association task and the co-occurrence statistics of natural language being different. In fact, all permutations of model comparisons of this kind are significant, except when comparing a full model with child-oriented and the adult-oriented networks to a restricted model with only the child-oriented network (Table 9). Thus, the child-oriented network explains variance that the adult-oriented network does not, but the opposite is not true.

**Table 9:** Model comparisons between networks

M_0_	M_1_	df	log*θ*_0_	log*θ*_1_	*χ*^2^	*p*	*p* (FDR)	BIC
*Adult*	Child	1	3090.528	3084.089	12.877	<.001	.001	6.573
	CHILDES	1	3090.528	3066.349	48.358	<.001	<.001	42.054
*Child*	Adult	1	3084.486	3084.089	0.795	.373	.435	–5.510
	CHILDES	1	3084.486	3060.235	48.503	<.001	<.001	42.198
*CHILDES*	Adult	1	3073.576	3066.349	14.455	<.001	<.001	8.150
	Child	1	3073.576	3060.235	26.682	<.001	<.001	20.377

**Fig. 6:**
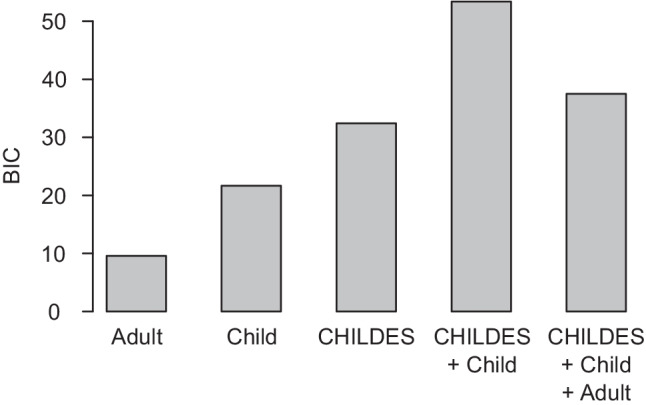
Incorporating network structure in addition to psycholinguistic factors using the preferential acquistion model. All BIC values reflect a comparison to the same restricted model; plus signs indicate that additional variables are added over the restricted model

Furthermore, if a full model that included growth values from all three networks (adult- and child-oriented word associations and CHILDES) is compared to a restricted network that excludes the adult-oriented network structure, predictions do not improve, (*χ*^2^(1) = 0.134, *p* = .715, BIC =  − 6.212). The BIC values obtained for various models involving the preferential acquisition growth values, from individual networks and combinations of networks, are summarized in Fig. 6.

## Discussion

The current study makes two complementary contributions. The first is to show that applying a cover story to the word association task elicits responses that differ from a standard (unconstrained) word association task. Consequently, the second contribution is a set of word association norms that appear to provide superior insight into the semantic environment of young children as indicated by improved models of vocabulary growth.

Our manipulation of the word association task instructions aimed to elicit child-oriented responses and was successful: responses in the child-oriented context were shorter and simpler words that tend to be acquired younger, and which are used more frequently and in more diverse contexts. Furthermore, the child-oriented word associations convey a different semantic structure than unconstrained (adult-oriented) associations and the co-occurrence statistics of child-directed speech (derived from CHILDES), and these differences account for unique variance in the typical trajectory of early word learning.

Dubossarsky et al. (2017) demonstrated that word association behavior changes over the life span, reflecting important individual differences in conceptual knowledge. These conceptual changes may imply age-related differences in the semantic environment. Their work implies that adult-oriented semantic norms should be relatively poor proxies for characterizing the child semantic environment. Although the previous literature focusing on early vocabulary acquisition has fruitfully used the University of South Florida’s word association norms (e.g., Bilson et al., 2015; Hills et al., 2009; Steyvers & Tenenbaum, 2005), more age-appropriate semantic norms will be necessary to detect the statistical and structural factors that children are sensitive to when acquiring language. Our findings agree with this suggestion; our child-oriented word association task provided important insight into the child semantic environment that could not be obtained from the adult-oriented word associations we collected, as indicated by the unique variance it explains in typical vocabulary development between 16 and 30 months of age.

The differences derived from our child-oriented word association task are particularly important given the emphasis of the learning environment associated with preferential acquisition. Our model comparisons revealed that the best model overall was obtained by jointly leveraging the network structure from child-oriented word associations and transcripts of child-directed speech (i.e., CHILDES). This multimodal approach to modeling early vocabulary growth is similar to the multiplex network modeling implemented by Stella et al. (2017), and is in keeping with recent evidence that each modality may have an advantage over the other when it comes to modeling different lexicosemantic variables (Nematzadeh et al., 2017; Vankrunkelsven et al., 2018).

### CDI-specific word association data

The CDI is one of the most widely used instruments in the field of child language development. Because of its ubiquity and because of open-science initiatives like WordBank (Frank et al., 2017), researchers may obtain vocabulary estimates for children with typical language development who vary from precocious early talkers to late talkers, as well as children with atypical language development associated with developmental disorders such as autism spectrum disorder (e.g., Colunga & Sims, 2017; Haebig et al., 2021; Jiménez et al., 2020). As such, one of the goals of our work was to acquire and publish word association norms for as many words on the CDI as possible using a protocol that makes those associations more age-appropriate than existing databases. Additionally, our datasets include disambiguated homophones (e.g., “orange [food]” and “orange [color]”). Although homonyms were excluded from the analyses reported in this work, this specificity is important because even young children develop subordinate word meanings as they grow in their lexical knowledge.

### Child-oriented word associations vs. child-directed speech

A natural reaction to this work is that, clearly, transcripts of adults interacting with real children is more valid than child-oriented word associations submitted by adults completing an online experiment alone. However, corpora of child-directed speech provide only a snapshot of input provided to a child. Indeed, a criticism of distributed semantic models based on text corpora is that the structure of semantic representations is heavily biased by the size of the text corpora and parameter tuning decisions (i.e., the grounding problem; Kumar, 2021; Kumar et al., 2022). To gather semantic data for the comprehensive list of words on the CDI, we also collected word association data by probing which words naturally go together after experimentally establishing a child-oriented context. To be clear, this work never intended to supplant or undermine the use of child-directed speech for studying language learning. The work was conceived while considering the prior success achieved using adult-oriented word associations to describe a semantic environment that may influence early language learning for children. We show that by manipulating the context in which adults provide word associations, those associations can become even more informative about the development of early vocabularies.

However, the question remains: why would one query word association data provided by adults in an attempt to describe the semantic environment of a child if transcripts of child-directed speech are available? Our results are consistent with prior work and discussions, indicating that each measure provides different but complementary perspectives on the child’s semantic environment. Word associations generated in our child-oriented task condition may reflect cognitive control processes that adults also rely on when modifying their language when speaking to children. Association tasks provide insight into the semantic environment *as encoded and retrieved* via mechanisms of learning and memory. Thus, semantic models derived from word association data tend to out-perform distributional models on predicting similarity judgments (De Deyne, Perfors, et al., 2016; De Deyne, Verheyen, & Storms, 2016b) and other behavioral rating of words (De Deyne et al., 2019).

We have replicated the well-established relationship between associative network structure, derived from free association data, and typical vocabulary development in children between months 16 and 30 via preferential acquisition. This relationship is strengthened when consulting our novel child-oriented association data and strengthened further when child-oriented associative networks and CHILDES co-occurrence networks are considered in tandem when modeling vocabulary growth. This aligns with recent work that indicates that semantic models based on distributional language statistics and semantic models based on word association data capture distinct and complementary information. For instance, semantic models based on word association data have been found to capture relatedness information (De Deyne, Perfors, et al., 2016) and visual and affective features of concepts (De Deyne et al., 2021; Vankrunkelsven et al., 2018). This is notable because recent research has indicated that statistical regularities in the visual domain and other visual features influence children’s early lexical development (e.g., Clerkin et al., 2017; Colunga & Sims, 2017; McDonough et al., 2011). Given this, it is possible that the word association data derived from the child-oriented task also capture aspects of the multimodal learning process that the *child* may experience, such as learning biases based on perceptual and affective features (e.g., Berman et al., 2013; McDonough et al., 2011; Perry et al., 2015; Perry & Samuelson, 2011). However, it is important to emphasize that this suggestion is only speculative (see Kumar et al., 2022 for a discussion about the utility of semantic network approaches for offering insight into both the structure of knowledge representation and the processes that are in play).

### Theories of lexical growth

Our work also contributes to an emerging consensus on how early vocabularies grow. Among the three models of network growth we considered, preferential attachment does not capture the process of word learning well. Instead, preferential acquisition appears to be most plausible of the accounts, after controlling for psycholinguistic variables, aligning with findings presented by Hills et al. (2009) that were based on a subset of nouns on the CDI, and on a larger set of words across other word classes (Hills, 2013). Preferential attachment and acquisition take divergent perspectives on the role of the learner and their relationship to the environment. Preferential attachment predicts that words that are central to the learner’s *internal* semantic environment—the relationships among words in their current vocabulary—drive what words will be acquired in the future. This attributes an active role to the learner, where their understanding of the world directs and filters their engagement with their environment. On the other hand, preferential acquisition predicts that the structure of the environment, regardless of what words are currently known, drives learning. This casts the learner in a more passive role, allowing the structure of the environment to impress itself upon them with less filtering and direction. However, this “passive” learner is not idle or disinterested—it is only by exploring the environment and appreciating its statistical regularities that learning can occur (Hay et al., 2011; Saffran et al., 1996).

We have juxtaposed preferential attachment and acquisition, but where does the “lure of the associates” fit in? This growth model is like preferential attachment in that it predicts that a child will be influenced by their known vocabulary when engaging with their environment. However, it is not the structure of their vocabulary that matters (as with preferential attachment), but how known words associate with unknown words. On this account, learning proceeds by luring in unknown words that associate with many known words. Consistent with prior work, our findings suggest that models based on the lure of the associates can significantly predict lexical growth (Hills et al., 2009; Hills et al., 2010).

Notably, regardless of which network the growth values are derived from, the correlation between the lure of the associates and preferential acquisition is high (*r* ≈ .53). Are lure of the associates and preferential acquisition equally plausible accounts of early vocabulary growth? Our results suggest no. When building models to predict when words typically enter the vocabulary, adding growth values generated via the lure of the associates to a model that already includes growth values generated via preferential acquisition (and the psycholinguistic baseline variables) does not improve model fit. However, when the order of inclusion is reversed, growth values generated via the preferential acquisition model do improve model fit. Thus, our analyses suggest that the most informative model of child vocabulary growth is preferential acquisition, and what is informative about the lure of the associates model is redundant with it. This echoes the original report by Hills et al. (2009).

### Limitations and future directions

The current study further enforced the importance of a child’s linguistic environment and presented additional data that can be used to examine word learning and word processing. Though the data that we report are compelling and complementary to the existing literature, it is important to note their limitations.

First, because we based our analyses on unweighted association networks constructed using the most liberal criterion for establishing connectivity between two nodes (any evidence of direct association or co-occurrence merited a directed edge), our network definitions may not have been optimal. Our protocol also revealed dramatic differences between network structures derived from CHILDES or word associations. The CHILDES network that we obtained by this protocol was very densely connected (∼40% of possible connections) while the adult- and child-oriented associations networks were sparsely connected (∼4% of possible connections). Increasing the co-occurrence threshold to 40 when constructing the CHILDES network achieves a level of sparsity on par with the networks derived from word associations. However, this splinters the environment into subnets with no paths between them and produced 105 orphan nodes with no connections at all. In fact, this splintering of the CHILDES network was found to begin immediately—merely increasing the threshold to two co-occurrences produced four orphan nodes. Previous work has also noted this all-or-none problem and the arbitrariness of co-occurrence requirements (see Castro & Siew, 2020, for a discussion on this topic). Meanwhile, the adult- and child-oriented association networks both afford paths between all 598 words studied in the environment, despite their sparsity.

It is also noteworthy that growth values estimated based on the CHILDES network were extremely colinear with the psycholinguistic baseline model (*R*^2^ = .906 for preferential acquisition). Indeed, without accounting for the psycholinguistic variables, models based on the CHILDES network and preferential acquisition were unable to predict language growth (*χ*^2^(1) = 0.109, *n*. *s*. relative to an intercept-only model). Thus, appropriate analysis of child-directed transcripts represents a methodological challenge. The structure is very different from adult-directed speech, let alone written media. While we are not the first to use the CHILDES transcripts in this way (Beckage et al., 2011; Hills, 2013; Hills et al., 2010; Huebner & Willits, 2018; Jimenez & Hills, 2017), there is room for future work to improve the protocol.

Second, the current study provides only limited insight into the cognitive mechanisms that underlie our successful manipulation of responses using our modified word association task. There are at least two alternative accounts: participants may have actively censored their free association process and reported only the child-appropriate responses (despite other words coming to mind more readily), or the context manipulation may have altered association strengths such that different associations dominated following the child-oriented cover story (e.g., Zeelenberg et al., 2003). While these alternatives are not mutually exclusive, appreciating the balance of these mechanics will be necessary to appreciate the value of context manipulations for more targeted semantic modeling. The effects of context on semantic access and related neurocognitive processes are being actively pursued on the frontiers of cognitive science (Hoffman et al., 2018; Jackson et al., 2021; Lambon Ralph et al., 2017; Yee & Thompson-Schill, 2016).

Third, we adopted a simplifying assumption that the optimal model parameters would be the same over the 16-to-30-month range and for all word types when estimating the probability that unknown words would be acquired at each month. Based on their own modeling work, Stella et al. (2017) proposed three learning stages during which vocabulary growth is best explained by different combinations of factors. Furthermore, Hills and colleagues note that words belonging to different syntactic classes may be primarily driven by different models of growth, with the lure of the associates being a compelling account particularly for nouns (Hills, 2013; Hills et al., 2010). Future work will dig deeper into these important nuances.

Finally, the word association studies we conducted presented participants with only early-age-of-acquisition words sampled from the CDI. Typically, word association tasks draw from a larger and more diverse sample of cues. It is possible (likely, we believe) that, over several association trials, participants infer a context that shapes their subsequent association behavior. Consequently, if a participant were to encounter the cue “dog” while completing two different word association studies, one sampling cues from the CDI and another sampling cues from thousands of frequently occurring words in adult language corpora, they might generate different responses. Indeed, the current study clearly demonstrates that association behavior is importantly context-sensitive. This consideration further motivated our decision to collect our own adult-oriented association responses, rather than drawing data from the SWOW or USF word association databases.

Furthermore, given the relatively small number of words on the CDI relative to words included in other larger word association databases (SWOW, USF), and given the strong effect of the child-oriented manipulation, the current child-oriented word association task could be used to gather semantic data in less frequently studied languages or dialects. Additionally, our task could prove to be useful for capturing cultural and dialectal variability (e.g., English in the USA, UK, Australia, African American English, Spanish in Mexico, Spain, Chile). The use of a child-oriented word association methodology could promote diversity and representation within the child language acquisition literature and could promote access to child language research.

In conclusion, the current study presents data from two word association tasks that yielded different associative responses. The child-oriented word association task not only yielded differences in the word responses’ age of acquisition, word length (number of letters, phonemes, and syllables), and contextual diversity, but also differences in semantic structure. Most importantly, we demonstrated that semantic networks derived from these child-oriented word associations support better models of a child lexical growth, suggesting that these networks are more in tune with the child semantic environment than those based on adult-oriented word association responses. These results suggest that it is possible to develop targeted semantic norms to better understand the experience of populations that are challenging or impossible to assess directly.

## Figures and Tables

**Fig. 7:**
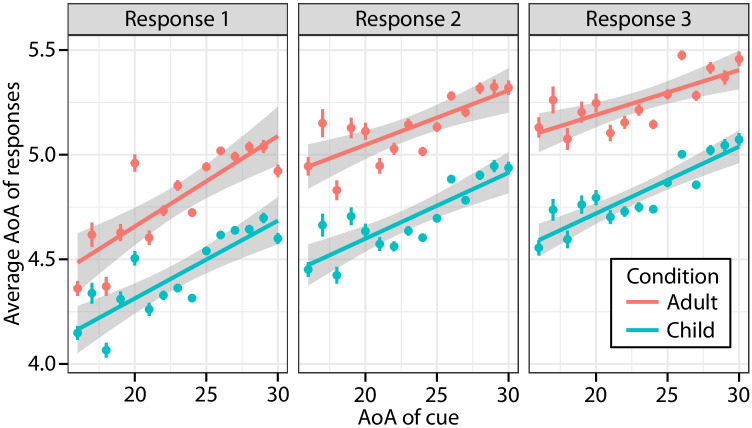
Relationship between the AoA of the cue and the mean AoA of the responses. Error bars reflect standard error of the mean. Best fit lines reflect separate linear models fit to each subset of the data. Error bands reflect the standard error of the slope

## Data Availability

The data and materials for all experiments are available at https://osf.io/3pmcw Code for replicating all experiments is available at https://github.com/crcox/CoxHaebig_BehavResMethods_2021 The netbuildr and netgrowr R packages developed in support of this work are available at https://github.com/crcox/netbuildr and https://github.com/crcox/netgrowr.

## Appendix 1 Excluded CDI Words

**Table:**

**Multiple meanings on CDI**	**No AoA**		**Multiple words**
Can (object) Can (verb) Chicken (animal) Chicken (food) Clean (action) Clean (description) Drink (action) Drink (beverage) Dry (action) Dry (description) Fish (animal) Fish (food) Orange (description) Orange (food) Slide (action) Slide (object) Swing (action) Swing (object) Watch (action) Watch (object) Water (beverage) Water (not beverage) Work (action) Work (place)	About Above An Babysitter Basement Before Beside But Camping Child Could Country Does Downtown Each Every Hate Hers If Into Last Much Naughty None Nurse Out	Person Play Pen Poor Scarf Snowsuit So Their Them Then Tights Tray Us Vagina Vanilla Walker Was Were When Which Wish Woods Would Yesterday Yourself	Babysitter’s name Child’s own name Give me five! Gonna get you! Pet’s name So big! This little piggy

## Appendix 2 Relationships between cue and response age of acquisition

It is possible that the effect of the child-oriented word association task manipulation is moderated by the age of acquisition (AoA) of the cue. Appendix Figure 7 below depicts the relationship between cue word AoA on the x-axis (as estimated from the Wordbank CDI database) and the AoA for the responses on the y-axis (as estimated from self-report norms collected by Kuperman et al. 2014) for each response index (response 1, 2, or 3 to a cue). In the figure, each point is an average over responses for cues of a particular AoA, and error bars reflect the standard error. Lines reflect a linear model with condition and response order as categorical factors.

The figure and simple linear model do not account for the dependencies in the data caused by each participant responding to multiple cues and cues being repeated across conditions. Thus, we constructed a linear mixed-effects model for statistical inference with random intercepts for participant and cue, random slopes for condition and response order by cue, random slopes for AoA of the cue and response order by participant, and fixed effects for condition, response order, and AoA of the cue. Response order was coded as a three-level factor with orthogonal polynomial contrasts (linear and quadratic trends). The model was fit using the MixedModels package v4.5.0 (Bates et al., 2022) in Julia v1.6.4 (Bezanson et al., 2017). Parameters are maximum likelihood estimates, and their standard errors are the square roots of the diagonal elements of the estimated variance–covariance matrix of the fixed-effects coefficient estimators. Reported z- and *p*-values are estimates based on dividing the parameter estimate by the standard error and referencing a standard normal distribution, respectively, which make simplifying assumptions about the distributions of these parameters.

The positive linear relationship between the AoA of the cue and the AoA of the responses is apparent in the figure and confirmed by the model (Appendix Table 10). The interaction between the AoA of the cue and condition is not significant, which suggests that the effect of condition is not moderated by the AoA of the cue. However, the three-way interaction between the AoA of the cue, condition, and the linear trend of response order was significant, meaning that the moderating effect of the AoA of the cue on the magnitude of the condition effect differs depending on whether we consider response 1, 2, or 3. Inspecting the simple effects by response order (Appendix Table 11), we see that the AoA of the cue and condition do not interact for response 1 or response 2, but the interaction is significant at *α* = .05 for response 3 (z = 2.16, *p* = .03). Despite the modest interaction at the third response, the condition effect is remarkably stable with respect to the AoA of the cue.

**Table 10:** Mixed-effects model

Fixed effects		*β*	SE	*z*	*p*-value		
	(Intercept)	0.00	0.01	0.27	.788		
	AoA_cue	0.07	0.01	6.23	<.001		
	Condition	−0.24	0.01	−26.13	<.001		
	R_L	0.16	0.01	22.20	<.001		
	R_Q	−0.03	0.00	−8.52	<.001		
	AoA_cue:C	0.01	0.01	1.25	.212		
	AoA_cue:R_L	−0.02	0.01	−2.24	.025		
	AoA_cue:R_Q	0.01	0.00	1.38	.168		
	C:R_L	−0.02	0.01	−2.84	.005		
	C:R_Q	0.01	0.01	1.65	.099		
	AoA_cue:C:R_L	0.01	0.01	2.30	.021		
	AoA_cue:C:R_Q	0.00	0.01	−0.71	.475		
Random effects		*σ*	Corr.				
*Participant*	(Intercept)	0.22					
	AoA_cue	0.01	1.00				
	R_L	0.07	0.71	0.71			
	R_Q	0.04	−0.31	−0.31	0.44		
	AoA_cue:R_L	0.01	0.11	0.11	−0.08	−0.39	
	AoA_cue:R_Q	0.02	−0.11	−0.11	−0.21	−0.29	0.98
*Cue*	(Intercept)	0.27					
	C	0.12	−0.33				
	R_L	0.16	−0.37	−0.05			
	R_Q	0.07	0.07	0.07	−0.75		
	C:R_L	0.08	−0.01	−0.13	−0.20	0.10	
	C:R_Q	0.05	0.06	0.00	0.06	−0.59	−0.37
*Residual*		0.91					

*Note.* The dependent variable is the AoA of the response. AoA of the response and AoA of the cue are standardized to have mean 0, standard deviation 1. Condition (C) is coded with adult-oriented −0.5 and child-oriented as 0.5. Response is polynomial contrast coded, evaluating linear (R_L) and quadratic (R_Q) trends over three responses. The model was fit to 343,178 observations, with 4079 participants and 598 cues

**Table 11:** Simple effects by response order

		Response 1		Response 2		Response 3	
Fixed effects		*β*	z	*β*	z	*β*	z
	(Intercept)	−0.12	−8.63	0.03	2.51	0.10	9.32
	AoA_cue	0.08	5.85	0.06	5.68	0.06	5.83
	C	−0.22	−21.62	−0.24	−23.64	−0.25	−23.02
	AoA_cue:C	0.00	−0.53	0.01	1.40	0.02	2.16
Random effects		*σ*	Corr.	*σ*	Corr.	*σ*	Corr.
PP	(Intercept)	0.19		0.22		0.24	
	AoA_cue	0.00	1.00	0.03	0.39	0.01	1.00
Cue	(Intercept)	0.34		0.27		0.24	
	C	0.16	−0.21	0.13	−0.39	0.12	−0.44
Residual	0.83		0.94		0.98		

*Note.* The dependent variable is the AoA of the response. AoA of the response and AoA of the cue are standardized to have mean 0, standard deviation 1. Condition (C) is coded with adult-oriented −0.5 and child-oriented as 0.5. Number of obs. response 1: 115,003; number of obs. response 2: 114,226; number of obs. response 3: 113,949
